# Rehabilitation effect of standing bed combined with early anti-gravity running table training on ankle fracture

**DOI:** 10.1038/s41598-024-52882-y

**Published:** 2024-02-13

**Authors:** JiaWei Chen, TianYu Wu, Shuigen Liu, Ying Guo

**Affiliations:** 1 Hunan Mechanical Electrical Polytechnic, Changsha, 410000 Hunan China; 2 The People’s Liberation Army Joint Logistic Support Force Sanya Rehabilitation and Recuperation Center, Sanya, 572000 Hainan China; 3 Department of Rehabilitation, Shenzhen Pingle Orthopedic Hospital and Shenzhen Pingshan Traditional Chinese Medicine Hospital, Shenzhen, 518000 Guangdong China; 4 Qiongzhong People’s Hospital of Li and Miao Autonomous County, Qiongzhong County, 572923 Hainan China

**Keywords:** Anatomy, Musculoskeletal system, Bone

## Abstract

To explore the clinical effect of standing bed combined with early anti-gravity running table training in the healing and functional recovery of anterior rotation external rotation ankle fractures. Fifty-two patients with ankle fractures of degree III or degree IV of PER admitted to Pingle Orthopaedic and Traumatology Hospital of Shenzhen City between September 2021 and January 2023 were selected for observation, and they were divided into 26 cases in each group according to the method of randomised numerical table into the control group and the observation group. The patients in the control group started the routine rehabilitation treatment on the 1st day after operation, and in the 0–2 weeks after operation, the affected limb was elevated and ankle pump training was carried out; in the 3–6 weeks after operation, joint mobility training, strength training and soft tissue release were carried out; and in the 6–8 weeks after operation, weight-bearing training was increased. The observation group added standing bed training on the 7th postoperative day and anti-gravity running table training on the 28th postoperative day on the basis of the treatment protocol of the control group. Bone density, ankle mobility and American Orthopaedic Foot and Ankle Society (AOFAS) ankle-hindfoot scores, pain, ankle mobility and swelling evaluations were compared between the 2 groups before and after 8 weeks of treatment, and the quality scores of bone scabs were compared between the 2 groups after 4 weeks of treatment. There was no statistical significance in the comparison of the items between the two groups before treatment (all *P* > 0.05), and the difference in the bone scab quality score was not statistically significant after 4 weeks of treatment (*P* > 0.05), and after 8 weeks of treatment, the bone scab quality score, bone mineral density and AOFAS scores, pain, ankle mobility, and evaluations were higher than those of the control group (all *P* < 0.05), and there was no significant difference in the degree of swelling (*P* > 0.05). Standing bed combined with early anti-gravity running table training applied to postoperative patients with PER III or IV degree ankle fracture can reduce the degree of pain and improve the ankle joint function.

## Introduction

The ankle joint is the most important weight-bearing joint in the human body and consists of the articular surface of the distal tibiofibula and the talus. Ankle fracture is the most common intra-articular fracture, which can lead to ankle deformity and activity limitation^1^. There are various types of ankle fractures, and the treatment and rehabilitation methods for different types of fractures are different, which have a greater impact on the postoperative functional recovery. Pronation-external rotation (PER) is an ankle fracture that occurs when the foot is in a pronated position at the time of injury and the talus is subjected to external rotation stress. Clinically, surgery is the main treatment for PER ankle fractures, but due to prolonged postoperative braking of the injured limb, joint adhesion and joint effusion may occur, affecting the function of the joint^2^; pain, swelling, dysfunction and other problems may remain in the later stage, and even cause traumatic arthritis, affecting the functional activities of the ankle joint^3^.

Some studies^4–6^ have reported that appropriate stress stimulation is conducive to promoting fracture healing, thereby accelerating the recovery process. However, the intensity of stress stimulation and weight-bearing time required by the fracture ends of patients at different times after surgery are different, so it is difficult to control the time and intensity of weight-bearing by conventional rehabilitation methods, and it is also impossible to monitor the real-time gait data of patients. The anti-gravity running platform follows the principle of gradual and individualised, adjusting the load on the ankle joint and the number of training sessions according to the fracture condition and the patient’s endurance to promote fracture repair^7^. The anti-gravity running platform training system can also be used to increase the stress stimulation of fracture breaks by real-time monitoring of the patient’s weight-bearing intensity, time, gait data, etc., and weight-bearing walking under visual feedback for postoperative fracture patients. Through reviewing the literature, the author did not see any relevant studies on the application of antigravity running table in the postoperative rehabilitation treatment of anterior rotation external rotation ankle fracture, so he conducted this trial. It is reported as follows.

## Information and methods

### General information

Seventy-eight patients admitted to Pingle Orthopaedic and Traumatology Hospital in Shenzhen during the period of September 2021–January 2023 with postoperative PER-type ankle fracture and hospitalised in the rehabilitation department of the hospital were selected for observation.Inclusion criteria: (1) clear history of trauma, according to the Lauge-Hansen (L-H) classification^8^, and preoperative diagnosis of PER III degree or IV degree ankle fracture by X-ray, CT examination, etc.; (2) unilateral closed injuries, history of two weeks or less, and no soft-tissue defects; (3) acceptance of postoperative rehabilitation training and follow-up, informed consent to this study and signature. Exclusion criteria: (1) patients with fracture of the anterior malleolus of the inner ankle; (2) ankle fractures accompanied by bone tumours, bone tuberculosis, chondrosarcoma and other pathological fractures; (3) age less than 14 years old, epiphyses have not yet closed; (4) patients with previous ipsilateral foot and ankle fracture or deformity; from which 52 patients were screened out using the method of random number table, the subjects meeting the inclusion criteria were numbered as No. 1–52, and entered into excel table, after applying the RAND function in excel sheet to generate random numbers, sorted from smallest to largest, after sorting 1–26 rows of subjects were assigned to control group and 27–52 rows were assigned to observation group.Fifty-two subjects were divided into an observation group and a control group, with 26 cases in each group. The baseline data of age, gender, body mass (BMI), intraoperative bleeding and other data of the two groups were compared, as shown in Table 1, and the differences were not statistically significant (*P* > 0.05) and were comparable. The study protocol was ethically approved by the Medical Ethics Committee of Shenzhen Pingshan Hospital of Traditional Chinese Medicine. The experiment complied with the Declaration of Helsinki, and the subjects were informed of the content and purpose of the study and signed a written informed consent form before the start of the study.

**Table 1:** Baseline data of postoperative ankle fracture patients in 2 groups.

Group	Sample size/case	Sex/case		Age/($\overline{x}$ ± *s*, years)	Surgical time/($\overline{x}$ ± *s*, minutes)	Bleeding volume/($\overline{x}$ ± *s*, ml)
		Male	Female			
Control group	26	10	16	39.73 ± 12.57	108.96 ± 14.79	270.38 ± 25.17
Observation group	26	12	14	40.73 ± 9.40	107.27 ± 10.32	267.16 ± 17.69
Test statistic		*χ*^2^ = 0.315		*t* = − 0.325	*t* = 0.479	*t* = 0.542
*p* value		0.575		0.747	0.634	0.590

### Treatment

All patients received routine treatment such as anti-infection, anticoagulation and analgesia after fracture surgery.

The control group receives conventional rehabilitation treatment methods: (1) 0–2 weeks after the operation, the postoperative fracture patients are educated, the affected distal limb is elevated as much as possible, the patients are instructed to carry out ankle pump training every day, and the ice is applied for 15 min immediately after the end of the training, and the inward contraction, abduction, forward flexion and backward extension exercises of the affected limb are encouraged, which mainly promotes the regression of ankle oedema, prevents thrombosis of the lower limb and muscle atrophy; the length of the treatment is about 60 min; (2) 3–6 weeks after the operation, at this stage, the plantarflexion, dorsiflexion, inversion and eversion joint mobility training of the ankle was emphasized to strengthen the strength exercises of the lower limb and the ankle joint in each direction of activity, and the joint loosening, muscle stretching and Pingle Guo’s Rongmiao kneading method were combined to promote the relaxation of the soft tissues around the ankle joint, and to further improve the ankle joint mobility; the length of the treatment was about 80 min; (3) 6–8 weeks after the operation, the treatment continued to be carried out as described above. Treatment, strengthen the strength training of the affected limb, continue to increase the ankle joint active and passive mobility exercises, and strive to increase to the normal joint range of motion, according to the X-ray examination of the fracture alignment healing, start crutches out of bed part of the weight-bearing training, and gradually increase the weight of the weight-bearing. The length of treatment is about 80–90 min, according to the patient feel slightly tired is appropriate.

The observation group added standing bed and anti-gravity running platform training (Tianjin Jin Wanxiang Medical Equipment Co., Ltd., Golden All Running Platform Training System, model: GA100S) on the basis of the treatment of the control group. The anti-gravity running platform training was carried out by the same group of skilled rehabilitation therapists, and patients were informed of the precautions related to running platform training before training.On the 7th postoperative day, the patient started standing bed weight-bearing training, the initial angle was 10 degrees, increased by 5 degrees every day, the frequency was once a day, each time for 30 min, 5 times a week, on the 28th postoperative day, the patient started anti-gravity running platform training, the first running platform training to lose 80% of the body weight, the patient’s two lower limbs stood on the running platform, and the upper limbs held the handrails to adapt to the sensation of weight loss, while avoiding excessive weight-bearing of the lower limbs, and on the second day, continued to lose 80% of the body weight. On the second day, the patient continued to lose 80% of his body weight and started to run on the running platform, the speed was set at 0.1 km/h, the gradient of the training was set at 0, and the training time was 30 min each time, 5 times a week; then the patient increased 8–10% of his body weight each week, and the walking speed was gradually accelerated until the affected side of the limb was fully weight-bearing. During the running platform training, patients can observe their lower limb movement status, stride length, both sides of the stride length and weight-bearing ratio through the screen in real time and make self-adjustment. The therapist needs to observe and guide the patient’s gait outside the transparent airbag, and remind the patient to keep up with the speed of the running platform and adjust the stride length in time. If the patient is older and there are safety factors, it is necessary to pay attention to ask the patient about the actual physical condition before training, and record the patient’s heart rate and blood pressure before and after training. If the blood pressure exceeds 180/110 mmHg, the heart rate exceeds 75% of the standard maximum heart rate, or there are symptoms such as dizziness, chest discomfort, etc., it is necessary to stop the training immediately and adjust the training volume for the patient.The initial training time was 10 min, then increased to 20 min, once/d, 5 times/week, depending on the patient’s adaptation and recovery.

**Figure 1:**
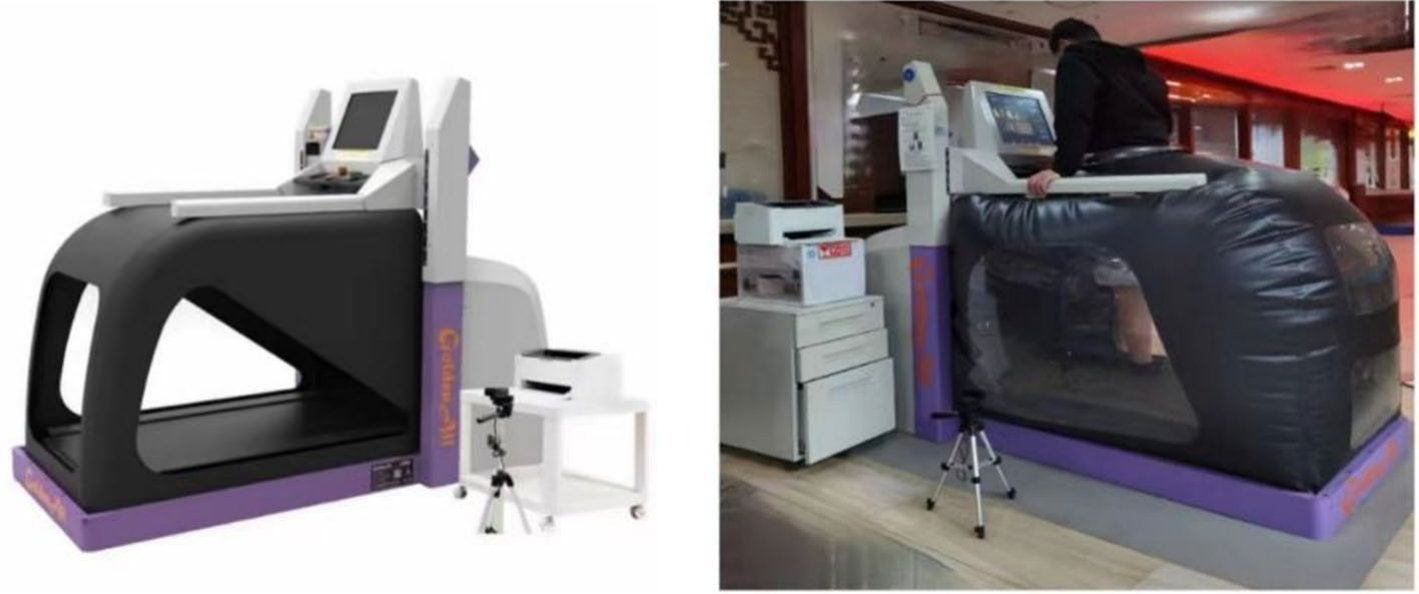
(The Anti-Gravity Running Table, also known as the Anti-Gravity Treadmill or Air Treadmill, is based on an inflatable cushion that allows the user to lose weight, using air pressure to gently lift the user and allow the user to weight-bear in the range of 20–100% of body weight for a comfortable weight-loss workout that is adjusted with an accuracy of 1%. Tianjin Jin Wanxiang’s Anti-Gravity Running Platform establishes a calibration system based on Differential Air Pressure (DAP) technology to provide zero-resistance gait and balance training with fall prevention support. The aim is to reduce the impact of gravity on the user’s running. In addition, the Anti-Gravity Running Table has many advantages in rehabilitation and sports training. Firstly, it reduces the impact of running on the joints and reduces the risk of injury. This is especially important for arthritis sufferers, recovering athletes and people in poor physical condition. Secondly, an anti-gravity running platform can help restore athleticism and speed up the rehabilitation process. By reducing the gravitational load, users can exercise more easily, building muscle strength and cardiorespiratory fitness. In addition, anti-gravity running table can also be used to train and improve runners’ speed, endurance and explosive power.).

The training cycle was 8 weeks, and efficacy was evaluated after 8 weeks. See Fig. 1.

### Evaluation indicators

#### Evaluation of fracture healing

(1) Bone scab quality score, bone scab quality score using the “Fernan-dezesteve radiological evaluation of bone scab grade standard”^9^, 4 weeks postoperative and 8 weeks postoperative comparison of the two groups of bone scab quality score, 0 points: no radiological bone scab at the end of the fracture; 1 point: cloudy bone scab at the end of the fracture; 2 points: bone scab formed on one side of the fracture end on both sides of the frontal lateral films; 3 points: bone scab on both sides of the fracture end on both sides of the frontal lateral films; 4 points: structural scab formation. Score: structural scab formation. (2) For bone densitometry, a dual-energy photon bone densitometer (HOLOGIC, USA, model Discovery-Wi) was used to detect the bone density of the proximal end of the tibia, which was reported in g/cm^2^, and the bone density of the proximal end of the tibia was compared between the two groups of patients on the 1st day of the postoperative period and at 8 weeks after the postoperative period.

#### Assessment of joint function recovery

Comparison of ankle function recovery at 1 day and 8 weeks postoperatively was assessed using the American orthopedic foot and ankle score (AOFAS), which consisted of 9 items including pain, maximum walking distance, ankle-posterior stability, and foot line of force, with a total score of 100 points and a minimum of 0 points.

#### Evaluation of pain, ankle mobility and swelling

(1) Pain score. The pain level of patients in both groups was assessed using the pain visual analogue scale (VAS) score at 1 day and 8 weeks postoperatively. The VAS score was 0–10, with higher scores indicating more severe pain. (2) Joint mobility evaluation. Active mobility of ankle dorsiflexion and plantarflexion was compared on postoperative day 1 and 8 weeks postoperatively using a joint angle measuring tape. (3) Evaluation of ankle swelling. On the 1st postoperative day and 8 weeks postoperatively, the circumference of the affected ankle joint was measured with a soft ruler (through the tips of the inner and outer ankle) and the normal circumference of the healthy ankle joint was measured, and the swelling value was calculated = circumference of the affected ankle joint—circumference of the healthy ankle joint.

### Data statistics

The data obtained were statistically analysed using SPSS25.0 software. 2 tests were used to compare the gender of patients in the 2 groups; all the measurement data including age, operation time, intraoperative bleeding haemorrhage were expressed as mean ± standard deviation (x ± s), and independent t tests were used for intergroup comparisons; intergroup and intragroup comparisons of the bone scab quality score, bone mineral density, ankle active mobility, and AOFAS scores were made using the paired-samples t-test. *P* < 0.05 was used to indicate that the difference was statistically significant.

### Informed consent statement

All methods were carried out in accordance with the relevant guidelines and regulations. All experimental protocols were approved by the Department of Rehabilitation, Shenzhen Pingle Orthopaedic and Traumatology Hospital (Shenzhen Pingshan District Hospital of Traditional Chinese Medicine). Informed consent was obtained from all subjects for all experimental protocols.

## Results

### Grouping results

A total of 52 patients were included in the study, 26 in the control group and 26 in the observation group.The baseline data of the patients in the two groups were comparable with no statistically significant differences (Table 1).

### Treatment results

#### Observe the healing of bone scab in the two groups of patients

Comparison of the quality score of bone scab of the 2 groups of patients after 4 weeks of treatment, the difference between the groups was not statistically significant (*P* > 0.05); after 8 weeks of treatment, the quality score of bone scab of the 2 groups of patients increased compared with the pre-treatment period, and the quality score of bone scab of the observation group was higher than that of the control group when comparing the two groups, and the difference was statistically significant (*P* < 0.05), see Table 2.

**Table 2:** Patient scab quality scores after ankle fracture surgery in 2 groups of patients.

Group	Sample size	Bone scab quality score ($\overline{x}$ ± *s*, points)		t value	*p* value
		4 weeks postoperatively	8 weeks postoperatively		
Control group	26	0.81 ± 0.40	1.31 ± 0.62	− 4.267	0.001
Observation group	26	0.77 ± 0.43	1.77 ± 0.71	− 6.142	< 0.001
t value		− 0.333	− 2.500		
*p* value		0.740	0.016		

#### Observe the bone density of the two groups of patients

Comparison of bone density of the 2 groups of patients on the 1st day after operation, the difference between the groups was not statistically significant (*P* > 0.05); after 8 weeks of treatment, the bone density of the 2 groups of patients increased compared with the pre-treatment period, and the bone density of the observation group was higher than that of the control group when comparing the groups, and the difference was statistically significant (*P* < 0.05), as shown in Table 3.

**Table 3:** Bone density of proximal tibia in patients after ankle fracture in 2 groups of patients.

Group	Sample size	Bone density ($\overline{x}$ ± *s*, g/cm^2^)		t value	*p* value
		1 day after surgery	8 weeks postoperatively		
Control group	26	0.55 ± 0.64	0.64 ± 0.08	− 3.910	< 0.001
Observation group	26	0.56 ± 0.53	0.74 ± 0.67	− 10.535	< 0.001
t value		− 0.142	− 4.856		
*p* value		0.888	< 0.001		

#### Comparison of postoperative AOFAS ankle-hindfoot scores between the two groups of patients

Comparison of AOFAS ankle-hindfoot scores between the two groups of patients at 1 day after surgery, the difference between the groups was not statistically significant (*P* > 0.05); after 8 weeks of treatment, the AOFAS ankle-hindfoot scores of the two groups of patients increased in comparison with the pre-treatment period, and the scores of the observation group were higher than those of the control group in the comparison between the groups (*P* < 0.05), as shown in Table 4.

**Table 4:** Comparison of patients’ AOFAS ankle-hindfoot scores after ankle fracture in the 2 groups ($\overline{x}$ ± *s*, points).

Group	sample size	AOFAS score ($\overline{x}$ ± *s*, points)		t value	*p* value
		1 day postoperatively	8 weeks postoperatively		
Control group	26	31.81 ± 4.93	75.73 ± 6.49	− 27.475	< 0.001
Observation group	26	31.31 ± 4.39	91.46 ± 6.09	− 40.871	< 0.001
t value		0.836	− 9.014		
*p* value		0.701	< 0.001		

#### Comparison of VAS score, ankle dorsiflexion and plantarflexion angles and ankle swelling between the two groups

There was no statistically significant difference between the VAS pain scores of the two groups at 1 day after surgery (*P* > 0.05); the VAS pain scores of the patients in the observation group were significantly lower than those of the patients in the control group at 8 weeks after surgery, and the difference was statistically significant (*P* < 0.05). The difference between the ankle dorsiflexion and plantarflexion mobility of the two groups of patients on the first day after surgery was not statistically significant (*P* > 0.05); the ankle dorsiflexion and plantarflexion angles of the patients in the observation group were significantly greater than those of the control group on the eighth week after surgery (both *P* < 0.05); and the difference between the ankle swelling degrees of the patients in the two groups was not statistically significant on the first day after surgery and the eighth week after surgery (both *P* > 0.05). Table 5 shows that the treatment of the observation group can effectively improve the ankle joint pain, dorsiflexion and plantarflexion angles, and there is no obvious advantage for the swelling degree compared with the control group.

**Table 5:** Comparison of VAS pain score, ankle dorsiflexion and plantarflexion mobility and ankle swelling between the two groups of patients ($\overline{x}$ ± *s*).

Group	n	1 day after surgery	8 weeks after surgery
VAS pain score (points)			
Control group	26	5.69 ± 1.32	2.27 ± 0.23
Observation group	26	5.43 ± 0.98	1.00 ± 0.31^a^
Dorsal extension mobility (degree)			
Control group	26	8.38 ± 2.51	13.73 ± 5.36
Observation group	26	8.08 ± 3.06	17.61 ± 4.72^a^
Plantar flexion mobility (degree)			
Control group	26	13.96 ± 2.14	32.35 ± 5.83
Observation group	26	14.35 ± 2.35	39.35 ± 5.86^a^
Joint swelling degree (degree)			
Control group	26	3.04 ± 0.67	0.83 ± 0.26
Observation group	26	3.18 ± 0.22	0.75 ± 0.35

Compared with the control group.

^a^*p* < 0.05.

## Discussion

The results of this study showed that after 8 weeks of continuous treatment, the bone scab quality score, bone density, AOFAS ankle-hindfoot score, pain, and ankle dorsiflexion and plantarflexion angles of the observation group on the affected side of the ankle joint were significantly better than the pre-treatment and the control group levels (*P* < 0.05), and only the joint swelling degree was not significantly different from that of the control group (*P* > 0.05), which indicated that the combination of the standing bed with the early antigravity running This indicates that the combination of early anti-gravity running training in the standing bed has a good promotion effect on the healing of anteriorly rotated externally rotated ankle fracture, which can effectively improve the patients’ postoperative motor function, improve joint mobility, and alleviate pain.

A Ankle fracture is a common joint traumatic disease in orthopaedics, and clinically mild ankle fractures without displacement can be treated conservatively; however, most patients need surgical repositioning to achieve precise anatomical repositioning and strong internal fixation, to restore the integrity of the articular surface of the ankle joint, and to avoid traumatic arthritis and secondary ankle instability caused by poor repositioning^10^. Prolonged postoperative braking of ankle fractures can lead to periarticular soft tissue adhesions and impaired ankle joint function, and about 10% of patients will have different degrees of impaired function after surgery^11^. Therefore, early postoperative functional exercises can avoid adhesion, joint stiffness and capsular contracture, maintain muscle tone and improve the quality of life of ankle fracture patients. There are many stable structures in the ankle joint, and the structure of ankle joint injury is diverse, the treatment and prognosis of different types and degrees of ankle fractures vary greatly, and it is difficult to exclude the influence of different factors on the postoperative functional recovery of patients. In addition, there is a lack of research on the functional recovery of PER ankle fractures after surgery. Therefore, in this study, we took the patients with PER ankle fracture as the observation object and analysed the effects of different postoperative functional rehabilitation exercises on the postoperative function.

Foreign scholars proposed^12,13^ that early weight bearing in postoperative ankle fracture patients can help accelerate fracture healing, and early postoperative weight bearing will not lead to problems such as loosening of internal fixation, and it is believed that early weight bearing training is advantageous for ankle joint function recovery and fracture healing. Some scholars have confirmed^14^ that early weight-bearing is beneficial to fracture healing, shortens fracture healing time, and promotes the recovery of joint function.The results of this study showed that early standing bed participation in weight-bearing training, and anti-gravity running table training intervention for 8 weeks after the increase in bone mineral density, the quality of bone scab score is higher than that of the control group who received conventional treatment, and there is no displacement of the fracture broken end and the phenomenon of loosening of the internal fixation, which suggests that early progressive weight-bearing can help to accelerate the healing of the fracture, which may be related to the stress stimulation of the fracture broken end, which is in line with the previous research findings Consistent. In this experiment, patients were instructed to start anti-gravity running table training from the 4th week onwards, and the results showed an increase in bone density at the 8th week, which was statistically significant compared with the pre-treatment period, proving that early weight-bearing has a promotional effect on the growth of bone density.It has been suggested that exercise training has a positive effect on bone mineral density^15,16^. Hejazi et al.^16^ in their study on the effect of exercise exercise on bone mineral density found that exercise significantly increased bone mineral density in the lumbar spine, femoral neck, and femoral rotor, which is consistent with the results of the present study.

The AOFAS Ankle-Hindfoot Rating Scale evaluates the functional recovery status of the ankle after ankle fracture in terms of pain, walking distance, walking on the ground, ankle stability and support ability, which can provide a more comprehensive understanding of the functional status of the ankle joint, and the higher AOFAS Ankle-Hindfoot Score predicts the better recovery of the ankle joint function^17^. Van’s team noted^18^ that antigravity running table training in patients with ankle fractures helps to increase lower limb muscle circumference and gait recovery. The results of the AOFAS ankle-hindfoot score in this study showed that the score of the experimental group was significantly higher than that of the control group, which suggests that early participation in antigravity treadmill training has a positive effect on the recovery of ankle function.Post-operative ankle fracture patients simulate the normal ground walking mode in advance under the reduced weight state of the anti-gravity running platform. During the training, the therapist sets the speed, slope, forward and reverse of the running platform according to the patient’s specific conditions, which can mimic the ground conditions in daily life in the author’s analysis, so that the patients can adapt to the normal gait training as early as possible, which then promotes the recovery of the functions, such as walking, ankle joint stability and support ability. The anti-gravity running table training not only provides patients with long-term, staged functional rehabilitation exercises, but also provides different anti-gravity training programmes for different patients based on the safety of the exercises and the actual situation of the patients, ensuring the feasibility of the rehabilitation training programme, preventing postoperative deformity, and maintaining the stability and mobility of the joints.

Postoperative activity limitation after ankle fracture is affected by a variety of factors, such as contracture of the soft tissues around the joint, formation of adhesive tissue, pain, and so on. The pain after ankle fracture is obvious, and at the same time, accompanied by damage to soft tissues such as muscles^19^, ligaments, tendons, etc., a large amount of plasma fibrous tissue exudes after the injury, and collagen fibres appear locally, leading to the formation of adhesions, and because the pain triggers the protective spasm of muscles, which leads to the reduction of joint movement, thus causing the limitation of ankle joint movement^20^. In this study, compared with the control group, the observation group had lower VAS pain scores at 1 day and 8 weeks after surgery, and greater ankle dorsiflexion and plantarflexion mobility after surgery, which indicated that the anti-gravity treadmill training could reduce the pain and improve the ankle mobility of the ankle joint patients. The reason for this is that early anti-gravity running table training makes up for the shortcomings in rehabilitation training, and can gradually increase the weight-bearing strength and time according to the patient’s ankle joint activity, and gradually increase the tolerance and maximum range of motion of ankle joint training through gait data acquisition, so as to improve the motor function of the ankle joint^21^. Studies have shown^22^ that early postoperative rehabilitation training for ankle fractures promotes faster blood circulation, less inflammatory factors around the injury, which in turn reduces pain, effectively relieves adhesions in the surrounding tissues, and improves joint mobility. In this study, there was no significant difference between the ankle joint swelling at each postoperative time point between the two groups of patients, but both were significantly improved after 8 weeks of treatment compared with 1 day postoperatively, suggesting that the rehabilitation modality of both groups had a good effect on reducing swelling. The reason for this was analysed as early rehabilitation measures after ankle fracture can help the recovery and regeneration of local blood circulation of the fracture, which is conducive to the repair of local soft tissues and the reduction of local tissue oedema^23^.

Early application of anti-gravity running platform system training should pay attention to the following points: (1) fracture site must have a strong internal fixation, to ensure that the fracture ends are fixed firmly; (2) in the up and down running platform moving and training must pay attention to the patient’s safety, there should be a person to accompany the patient, to prevent falls; (3) gait training process according to the patient’s own situation to increase the affected limbs of the weight-bearing force and walking speed, and gradually increase the affected limb muscle strength and ankle mobility; (4) during the treatment period to follow the gradual and progressive, gradual incremental principle, do not be too eager to get quick results and blindly increase the amount of.

### Limitations

The limitations of the results of this study are as follows: (1) Only 52 subjects were included. Despite the differences between individuals, the subjects were carefully selected from 78 patients with similar age and lesion conditions; (2) the subjects’ pre-loss exercise habits and exercise levels were not compared, and their influence on the experimental results was not considered; (3) tests were only performed on the knee at 1 day postoperatively, and at 8 weeks post-treatment, and the next step of testing was not performed at a later stage to determine the effects of antigravity treadmill training on the functional and stability of the ankle joint; (4) this study only investigated the healing and functional recovery of anterior externally rotated ankle fractures and did not explore other types of ankle fractures, so the study is limited; (5) the sample size of this study is limited, and the collection of clinical data is limited by the researcher’s own clinical ability, which may have a certain impact on the final results. Therefore, it is necessary to carry out relevant basic biomechanical studies in the future to further explore.

Standing bed combined with anti-gravity running table training and conventional rehabilitation treatment had different degrees of efficacy, but the efficacy of standing bed combined with anti-gravity running table training was significantly better than that of conventional rehabilitation treatment, especially for the improvement of dorsiflexion function of the ankle after PER ankle fracture, which promotes the recovery of the function of the ankle fracture at the later stage, and provides a new idea for the clinical treatment of the dysfunction of the ankle fracture of the PER after the operation.

## Data Availability

Due to the nature of this study, participants in this study did not agree to share their data publicly, so no supporting data are available. Data can be obtained from the corresponding author upon reasonable request.
